# An Anatomical Thermal 3D Model in Preclinical Research: Combining CT and Thermal Images

**DOI:** 10.3390/s21041200

**Published:** 2021-02-09

**Authors:** Franziska Schollemann, Carina Barbosa Pereira, Stefanie Rosenhain, Andreas Follmann, Felix Gremse, Fabian Kiessling, Michael Czaplik, Mauren Abreu de Souza

**Affiliations:** 1 Department of Anesthesiology, University Hospital RWTH Aachen, Pauwelsstrasse 30, 52074 Aachen, Germany; cbarbosapere@ukaachen.de (C.B.P.); afollmann@ukaachen.de (A.F.); mczaplik@ukaachen.de (M.C.); 2 Institute for Experimental Molecular Imaging, University Hospital RWTH Aachen, Pauwelsstrasse 30, 52074 Aachen, Germany; srosenhain@ukaachen.de (S.R.); fgremse@ukaachen.de (F.G.); fkiessling@ukaachen.de (F.K.); 3 Post Graduate Program on Health Technology, Polytechnique School, Pontifical Catholic University of Paraná, R. Imac. Conceição, Curitiba 1155, Brazil; mauren.souza@pucpr.br

**Keywords:** thermal imaging, CT, 3D model, VisualSFM, camera calibration, registration, image processing, SfM, multimodality, 3D THERMO-SCAN

## Abstract

Even though animal trials are a controversial topic, they provide knowledge about diseases and the course of infections in a medical context. To refine the detection of abnormalities that can cause pain and stress to the animal as early as possible, new processes must be developed. Due to its noninvasive nature, thermal imaging is increasingly used for severity assessment in animal-based research. Within a multimodal approach, thermal images combined with anatomical information could be used to simulate the inner temperature profile, thereby allowing the detection of deep-seated infections. This paper presents the generation of anatomical thermal 3D models, forming the underlying multimodal model in this simulation. These models combine anatomical 3D information based on computed tomography (CT) data with a registered thermal shell measured with infrared thermography. The process of generating these models consists of data acquisition (both thermal images and CT), camera calibration, image processing methods, and structure from motion (SfM), among others. Anatomical thermal 3D models were successfully generated using three anesthetized mice. Due to the image processing improvement, the process was also realized for areas with few features, which increases the transferability of the process. The result of this multimodal registration in 3D space can be viewed and analyzed within a visualization tool. Individual CT slices can be analyzed axially, sagittally, and coronally with the corresponding superficial skin temperature distribution. This is an important and successfully implemented milestone on the way to simulating the internal temperature profile. Using this temperature profile, deep-seated infections and inflammation can be detected in order to reduce animal suffering.

## 1. Introduction

Several scientific and medical advantages are based on findings from animal trials, which have led to significant improvements in healthcare and quality of life. However, although these experiments are still considered useful in certain research fields, they trigger discussions regarding the animals’ well-being [1]. To ensure animals’ welfare, the 3R principle (replacement, reduction, and refinement) was introduced [2]. One aim of the 3R in particular is to replace animal trials with alternatives whenever possible. On the other hand, this principle recommends a reduction of the number of laboratory animals and the refinement of the trials by minimizing stress and pain [3]. Animal experiments are also used to improve the understanding of inflammatory diseases due to their multifactorial etiologies [4]. Therefore, it is also important to detect inflammation and infections early and efficiently to reduce the animals’ stress. Unfortunately, there are no accurate and objective tools or methods able to define the characteristics of deep-seated inflammation and infections or monitor their courses over time in animals undergoing scientific procedures. Nowadays, experimental procedures are categorized according to their severity, and the suffering level of each animal is assessed subjectively by the researcher.

Due to the improvements of thermal cameras in recent years in terms of resolution, size, and price, the research interest in thermal imaging grew [5]. This modality enables the measurement and detection of a scene’s temperature distribution [6]. From engineering to medical approaches, the measurement of the outer information with the aid of thermal imaging has various fields of application [7]. Due to its noninvasive and passive characteristics [3], infrared thermography is a suitable modality for animal-based research: It reduces both the possible spread of superficial infection based on touching the subject and the stress of the animals [8]. Beyond that, this technique is capable of capturing one of the cardinal symptoms of infection: calor (heat). Laboratory animals can be monitored remotely and inconspicuously by infrared thermography, for example, to assess stress, motion activity, and wound infection [9]. In addition, pathologies that lead to an altered surface blood flow can also be measured [10]. Temperature changes—for example, due to hypertension heart disease [11], inflammation [12], and fatty liver disease [13], or rheumatoid arthritis [14]—can be detected using thermal imaging. At the same time, local inflammation can be attributed to a tumor, which might lead to an altered local body temperature compared to the healthy tissue as a side effect due to abnormal metabolic and perfusion rates [15].

To quantify inflammation and to improve diagnostics and monitoring, a thermal 3D scan model for human medicine was introduced by Chromy and Klima [5], calculating a 3D view of the acquired 2D thermal images and a 3D scan of an injured toe. Similar approaches were followed by Grubišiü and Boš [7] and van Doremalen [16] for diabetic foot disease, presenting the development of a system combining an active scanning system and a thermal camera. These approaches calculate a 3D model of the thermal images. De Souza [17] went one step further and combined the thermal images with magnetic resonance imaging (MRI) data in 3D for a dentistry application.

This paper presents a new approach to generate anatomical thermal 3D models combining both the inner and outer information from thermal images and CT data for preclinical research. Instead of one planar thermal image showing only one particular angle and a limited area, multiple images are used to create a thermal 3D shell. This shell allows a 3D analysis of the temperature distribution of the animal and is then combined with the anatomical information of the CT data. Medical imaging systems such as CT or MRI provide an animal’s anatomical information and form the gold standard in preclinical research. Compared to MRI, CT offers the advantages of faster acquisition and scanning times, better contrast, and simpler and more cost-effective settings, which is why CT data recorded within an ongoing animal trial were used in this paper. The inclusion of inner information will enable the improvement of evaluations of the state of the animals’ health in the future.

The generation of anatomical thermal 3D models serves as an essential milestone for a simulation approach to calculate the inner temperature profile based on these models. With the help of such a simulation, conclusions about internal infections, inflammation, and other pathologies could be drawn from the superficial temperature distribution.

## 2. Methodology

**Figure 1:**
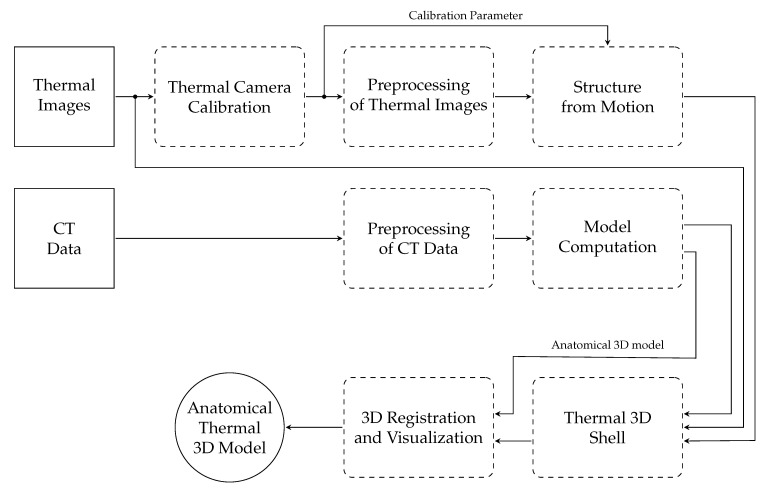
Block diagram of the anatomical thermal 3D model generation process.

The process to generate anatomical thermal 3D models is composed of several working steps (Figure 1). To facilitate understanding of the methodology, the most important designations are:

- Point cloud: 3D point cloud of the thermal images generated with the structure from motion (SfM) algorithm.
- 3D CT shell: Outer 3D shell based on CT data.
- Anatomical 3D model: 3D inner information based on CT data.
- Thermal 3D shell: 3D shell computed using the thermal images, point cloud, and 3D CT shell.
- Anatomical thermal 3D model: 3D combination of inner information (anatomical 3D model) and outer temperature distribution. (thermal 3D shell): anatomical 3D model + thermal 3D shell.

In the first step, data from both modalities (thermal imaging and CT) were recorded. Afterwards, the parameters of the thermal camera were determined via camera calibration. The third step consisted of preprocessing of the thermal images and calculating their point cloud using structure from motion. The CT data were also preprocessed and with the help of model computation both the 3D CT shell and the anatomical 3D model could be determined. Thereafter, the thermal 3D shell was computed by wrapping the thermal images around the 3D CT shell using this point cloud. The 3D registration of the thermal 3D shell and the anatomical 3D model, and visualization using the 3D THERMO-SCAN method [18], formed the final step to generate anatomical thermal 3D models.

### 2.1. Thermal Camera Calibration

**Figure 2:**
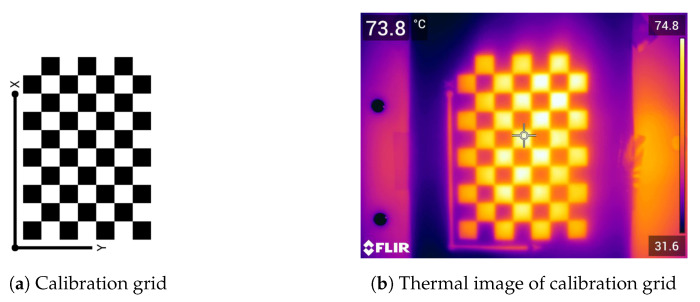
Camera calibration. (**a**) Original camera calibration grid and (**b**) an example of a calibration grid measured with infrared thermography (using a heat lamp).

Calculating the camera parameters was necessary to improve the process of generating the anatomical thermal 3D model and the overall results. For this purpose, a checkboard pattern with white and black squares, typically used for visual cameras, was required to calculate the intrinsic parameters. Taking advantage of the emissivity factors of darker surfaces compared to lighter surfaces [7], this pattern demonstrated suitability for thermal camera calibration when heated with a heat lamp. The original calibration pattern from MATLAB (The MathWorks, Inc., Natick, MA, USA) was used (see Figure 2a) and was fixed to a solid background to avoid distortion. An example of a resulting image is shown in Figure 2. Therefore, the calibration grid could be detected within a sequence of images with the same distance as for the thermal mouse data using the Camera Calibrator from MATLAB. The parameters extracted by the calibration (e.g., focal length, optical center point, and radial distortion) were used in the SfM algorithm to increase the number of feature points and improve results.

### 2.2. Preprocessing of Thermal Images

Preprocessing was required to generate the thermal 3D model. First, the data from the thermal video were transformed to single frames. To reduce computational cost and avoid redundancy, only every 10th frame was considered. The amount of feature points (distinct points within the image that are characterized by significant temperature changes or edges) had to be increased to improve point cloud creation. In contrast to other regions, there were few to no feature points within the abdominal area of the mouse. Further (1) contrast enhancement of the images and (2) improvement of the extrinsic landmarks (by attaching extrinsic landmarks on the abdominal region of the mouse and preprocess them using a computer vision algorithm) were required to improve the SfM algorithm.

**Figure 3:**
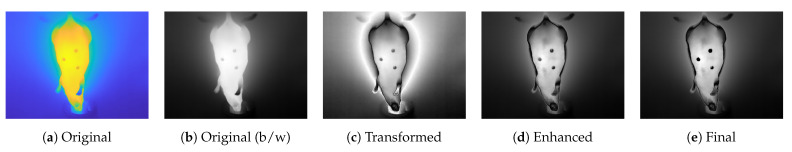
Examples of preprocessing: Contrast enhancement and extrinsic landmarks. While (**a**,**b**) represent original image data (in color and black-and-white), (**c**) shows the result after contrast enhancement to [19]. (**d**) The contrast enhancement improvement for our approach and the improvement of extrinsic landmarks, and therefore, the final preprocessed image in (**e**).

Regarding the enhancement, the intensity transformation presented by Krefer et al. [19] was applied (Figure 3c). Compared to the original black-and-white image (Figure 3b), the contrast within the mouse region of interest (ROI) increased, but the background became inhomogeneous. Although this would lead to more feature points in one particular image, the overall result would deteriorate due to high intensity changes in the background, which led to different locations of those feature points between multiple images. A similar result was obtained when trying to separate the mouse from the background. This was not effective because of the radiation of the body temperature to the background; this led to different segmentations at the individual images, which degraded the SfM approach. In summary, the mouse ROI was improved by the intensity transformation, but the background from the original was more consistent. For this reason, the minimum temperature for each pixel of Figure 3b,c was calculated within an iterative process, combined with a histogram equalization function from MATLAB to enhance contrast within the ROI and at the same time uniform the background (see Figure 3d).

Extrinsic landmarks are frequently used for registration of different image modalities [20]. In this case, they were used for landmark-based registration between subsequent images. Based on previous studies and on empirical knowledge, a homogeneous temperature within the abdominal region was expected. In thermal imaging, homogeneous temperature distribution means very low contrast and thus a lack of feature points, which are essential for the SfM algorithm. To solve this problem and improve registration robustness, 3 extrinsic landmarks were attached to the mouse. Since the temperature difference between these and body temperature was significant (see Figure 3a), the thermal images presented a higher contrast and dense feature points could be found within the ROI. To increase the amount of feature points found in the area of the extrinsic landmarks, a semi-automated algorithm was implemented. The positions of the landmarks within the thermal images were determined manually by user input. Starting from this marker, a region-growing algorithm was implemented to segment the whole area of each landmark. The result of this algorithm was a binary mask that can be used to set the intensity within the extrinsic landmarks to an equal value matching each image. It permitted us to homogenize the intensity of the extrinsic landmarks within the thermal image. Therefore, the correlation between these areas among the images was higher.

**Figure 4:**
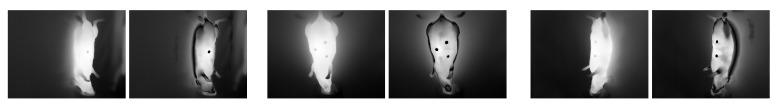
Examples of images after preprocessing: Contrast enhancement and extrinsic landmarks. (**Left**): original image. (**Right**): preprocessed image.

The result of the whole preprocessing process, including contrast enhancement and extrinsic landmark improvement, is displayed in Figure 3e. Figure 4 shows the differences between the original and preprocessed images with 3 examples, shown in black and white to illustrate the change in contrast.

### 2.3. Structure from Motion

**Figure 5:**
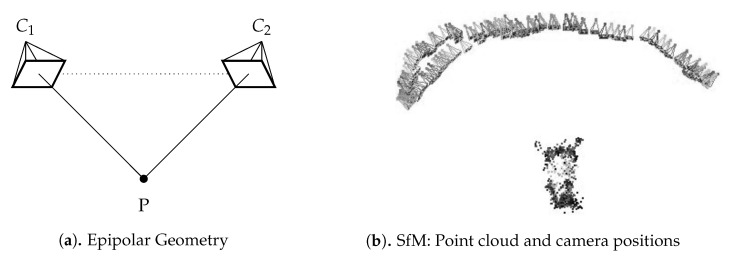
Structure from motion algorithm: (**a**) schematic view of epipolar geometry, (**b**) point cloud and camera positions of mouse M1.

Using multiple planar images as input, the SfM algorithm was applied to generate a 3D point cloud from the set of thermal camera images. An underlying theory behind this approach is epipolar geometry, which describes the relationship of a feature point (P) in each image ($C_{1}$,$C_{2}$) [21] (see Figure 5a). Therefore, the more feature points are found in each image and the more these feature points correspond across images, the better the result. For this step, the SfM approach was utilized using the VisualSFM GUI application Open Source by Changchang Wu [22]. This consists of a set of techniques that calculate a 3D point cloud based on a compilation of 2D images collected around the object to be inspected. In addition to the preprocessed thermal images, some camera information based on the previously described camera calibration was needed for this modeling process, such as focal distance, principal point, and radial distortion. Within VisualSFM, the “Used Shared Calibration” and “Use file title as Identifier” three settings were selected. “Set Maximum Dim” was set to 4096, and the setting option “Search Multiple Models” was deactivated. The SfM method involves calculating not only the coordinates of the object in 3D space but also the camera’s positioning [17]. Figure 5b illustrates the generated 3D point cloud and the camera positioning based on the data of mouse M1.

### 2.4. Preprocessing of CT Data and Model Computation

**Figure 6:**
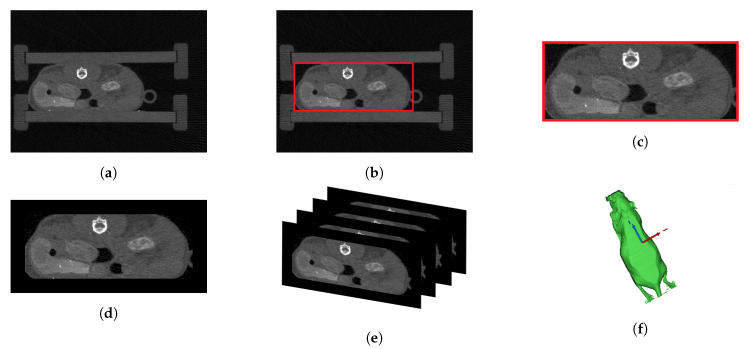
Preprocessing of the CT data: (**a**) original CT image, (**b**) original CT image with cutting algorithm box, (**c**) trimmed image, (**d**) final CT slice with a black border to provide a uniform surrounding area, (**e**) overlaid visualization for 3D modeling, and (**f**) 3D CT shell.

CT uses mathematical reconstruction of x-rays to display inner information by creating a set of anatomy sectioned into image slices, covering the whole body under study. This approach allowed the generation of 3D models of the body [17]. As shown in Figure 6a, the anatomy data consisted of several CT image slices. Each slice provided inner anatomical information based on its position within the sequence. To avoid any movement during the acquisition, the mouse was placed into a narrow box on its belly.

To generate the 3D CT shell of the mouse, a background segmentation of its body was performed using the thresholding tool in Mimics version 17.0 (Materialise NV). Afterwards, it was necessary to remove the surrounding box from the CT data (see Figure 6a). For this purpose, a cutting algorithm was used together with multiple slice edit in Mimics (represented by the red box in Figure 6b,c). Then, all the images were pilled up and connected forming a 3D model in the space (Figure 6e,f). On the other hand, to generate the anatomical 3D model, the slices after the cutting algorithm were used (Figure 6c). Additionally, a black border was added to provide a uniform region surrounding all image slices (Figure 6d). Both the 3D CT shell (Figure 6f), and the anatomical 3D model containing the individual anatomical slices’ information, which were also pilled up forming the inner representation for the anatomical approach, were determined based on the same CT data. Therefore, Figure 6 represents not only the preprocessing of the CT data (Figure 6a–d), but also the model computation (Figure 6e,f).

### 2.5. Thermal 3D Shell

**Figure 7:**
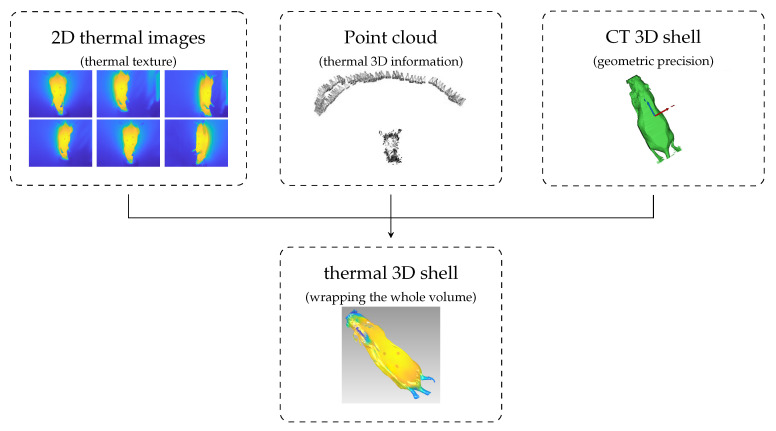
Representation of Thermal Outer Shell Showing 2D Infrared Images and 3D External Surface.

**Figure 8:**
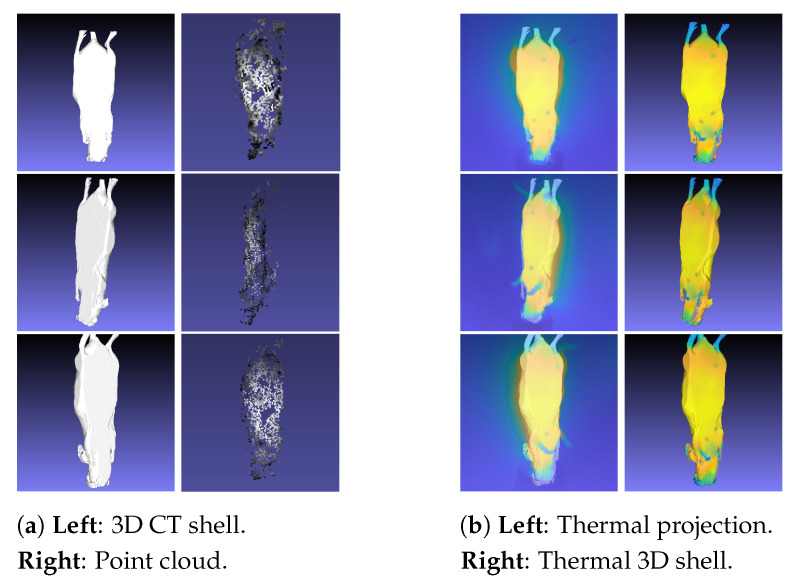
Generation of the thermal 3D shell: (**a**) Alignment between the 3D CT model and the point cloud. (**b**) Thermal image projection to generate the thermal 3D shell.

This section explains the methodology used for the alignment between the point cloud, the thermal images, and the 3D CT shell and the associated creation of the thermal 3D shell (see Figure 7). At this stage, there were 2 separate steps: (1) alignment between the 3D CT model and the point cloud, and (2) thermal imaging projection (Figure 8). Both steps were performed in MeshLab, an open-source system developed by the Institute of Information Science and Technology of the Italian National Research Council.

The alignment consisted of a coordinate system transformation. A redimensioning of the outer 3D model was employed according to the real size of the object to be imaged. Here, the manipulators tool function was used with the options rotate, translate, and scale. The camera calibration provided the correct dimensions of the object in terms of including scale and appropriate metrics. Therefore, we also included the camera’s positioning and the obtained calibration information in this transformation.

After the alignment process, projection of the infrared images onto the 3D CT shell was performed. Based on the point cloud information, the thermal images were wrapped around the 3D CT shell to generate the thermal 3D shell (Figure 8). Therefore, the Raster Mode (RM) was applied. RM superimposed the thermal images over the 3D model for inspection, which allowed synchronization between this 3D model and the original thermal images. The respective pixel value in 3D was calculated as a weighted average for the relevant camera positions, depending on the distance of the point to the camera and the distance to the center of the image [19]. Additionally, in MeshLab, we used the texture filter with the function parametrization + texturing from registered rasters. This final approach allowed the generation of a thermal 3D shell with the thermal images already incorporated into the 3D CT shell. Then, the next step is the registration itself between the 3D thermal shell and the anatomical 3D model, containing the inner information.

### 2.6. 3D Registration and Visualization

**Figure 9:**
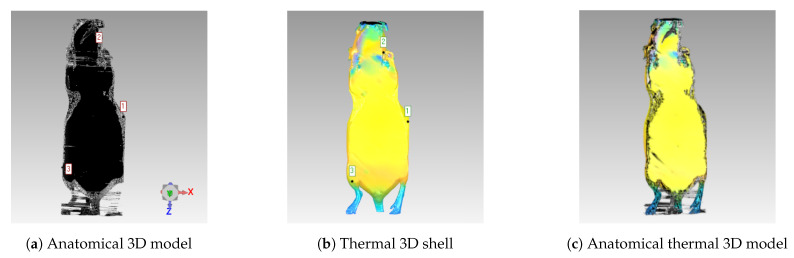
Registration Process: (**a**) 3D model generated from anatomical CT images. (**b**) Thermal 3D model. (**c**) Registration of both 3D models, generating the complete model (visualized using the same coordinate system).

After combining the thermal images using the point cloud with the 3D CT shell, the next step was to register the anatomical 3D model (inner CT information) with the thermal 3D shell using an affine registration with Geomagic Wrap version 12.0 (3D systems, Inc.). Here, the main focus was related to the coordinate system transformation, in order to unify and enable the 3D visualization at the same system. A registration was performed between the 3D thermal shell and the CT data (where the image slices are placed) because the originally generated 3D thermal shell was placed using a different reference system (due to the different acquisition methods). The camera positions were essential for creating the thermal 3D shell and also were included in the system of thermal images. For this reason, the 3D CT shell had to be transformed into the thermal image system to be registered with the thermal images. Therefore, the reference system regarding the thermal 3D shell was the infrared camera. On the other hand, for the registration of the inner information, the reference system was the actual tomography. It was essential for the visualization tool that the individual CT slices not be transformed. This justified the back transformation of the thermal 3D shell into the reference system of the CT data. To illustrate this coordinate system transformation, initially the thermal 3D shell (Figure 9b) was manually transformed to the anatomical 3D model (Figure 9a), which enabled both imaging modalities to be visualized together (Figure 9c). The registration was then refined within Geomagic Wrap using the global registration algorithm, which employed a best-fine registration method—in this case, the iterative closest point (ICP) algorithm [23]. Thus, the registration process (illustrated in Figure 9) represented this coordinate system transformation, in order to unify all of the data into a common reference system.

The last step was the 3D THERMO-SCAN visualization interface [18], which visualizes both imaging modalities altogether. The software for visualization was developed for Microsoft Windows using C++ and MATLAB. 3D visualization was performed using Open Graphics Library (OpenGL) version 1.4, OpenGL Utility Library (GLU), and OpenGL Utility Toolkit (GLUT) libraries. The PLY file format was used to describe the 3D object as a polygonal model. Here, the special feature is that different areas of the model can be selected by the user, and individual CT information can be displayed with the surrounding thermal information. This 3D visualization interface enables inner and outer inspection, allowing representation from not only the axial, but also from the coronal and sagittal slicing representations.

## 3. Experimental Protocol

**Figure 10:**
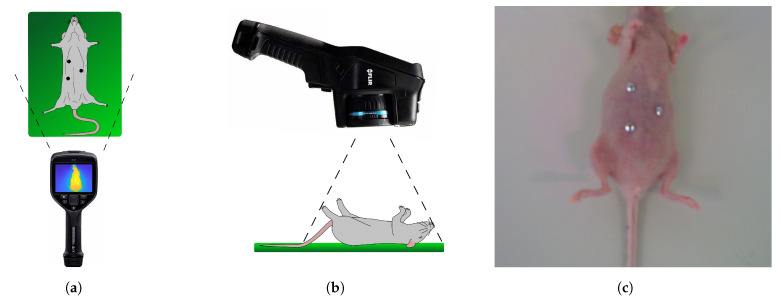
Schematic view of data acquisition: (**a**) Top view (with extrinsic landmarks), (**b**) side view and (**c**) visual image (with extrinsic landmarks).

Thermal images and CT data were recorded at the Institute for Experimental Molecular Imaging of RWTH Aachen. Using an ongoing animal trial (AZ84-02.04.2016.A076), recordings of 3 female SKH1 mice from the institute’s own breeding were acquired and analyzed. Data acquisition was scheduled during the trial’s regular anesthesia to reduce the animals’ stress and meet the 3R principle’s requirements. Thermal video was recorded using a FLIR E95 thermal camera (FLIR Systems Inc., Wilsonville, OR, USA) with a resolution of 464 × 348 px and a thermal sensitivity better than 0.04 °C at 30 °C. For subsequent thermal image registration, extrinsic landmarks were placed on 3 different locations on each mouse’s skin (see Figure 10a). As these landmarks were attached right before the video recording, they appear cold compared to the mouse’s skin temperature and are easily visible in the thermal images.

The camera was hovered around the mouse at a distance of 30 cm (see Figure 10). Therefore, the video shows multiple angles of the mouse focused on the abdominal area. In order to avoid thermal variations, the data collection was performed as quickly as possible: for the mice in this research, it took less than 1 min for the thermal data collection.

The CT data, which were regularly recorded in the ongoing animal trial with the U-OI system (MILabs B.V., Houten, The Netherlands), were also provided by the Institute for Experimental Molecular Imaging of RWTH Aachen.

## 4. Results

**Figure 11:**
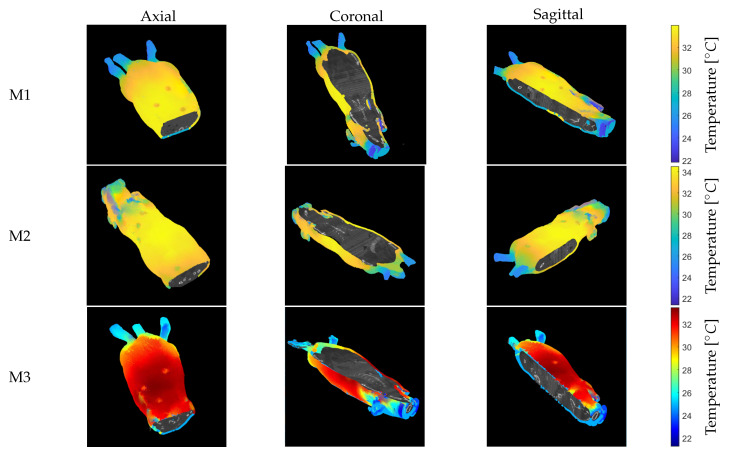
Anatomical thermal 3D model for mice M1–M3. The color map of M3 was changed to demonstrate a possible adaptation, which can be helpful for manual analysis of the temperature distribution. (**Left**): axial. (**Middle**): coronal. (**Right**): sagittal.

Anatomical thermal 3D models of 3 anesthetized mice (M1–M3) were created using this methodology. The model consists of a thermal 3D shell combined with the anatomical information based on the CT data inside the model (Figure 11). Note that the three cross-sectional slices are visualized: axial, coronal, and sagittal. Video S1 (Supplementary Material) (M2) and Video S2 (Supplementary Material) (M3) provide short animations showing the final anatomical thermal 3D model displayed in the 3D THERMO-SCAN visualization tool. It should be noted that the color palette can be varied within the process of generating anatomical thermal 3D models in order to enable an individual thermal analysis depending on the application.

Camera calibration and image processing tools were implemented to improve the results and stabilize the process. Regarding VisualSFM, some parameters allowed comparison of the point cloud creation results of different input image data sets: Depending on the input data, VisualSFM does not necessarily use all input images as camera positions. Therefore, the number of images used (camera positions; see Table 1) changes based on the input data. Other parameters are the number of points in the point cloud (points; see Table 1) and the associated number of projections (projections; see Table 1); the latter describe the connections between actual 3D points within the point cloud and the image planes of the respective cameras. Accordingly, the number of projections illustrates how often the points of the point cloud were detected as features by multiple cameras. Table 1 shows the comparison of the previously described parameters for the preprocessed images with and without added camera information compared to the original images.

## 5. Discussion

In this research, the process of generating an anatomical thermal 3D model by combining outer temperature distribution with inner anatomical information was implemented and adapted for the acquired mouse data. The three anatomical thermal 3D models we generated generally showed accurate temperature distribution regarding the abdominal area of the mice. This proof of concept is an important and necessary step for a later simulation of the thermal properties of inner tissue based on the temperature distribution of the skin.

Although VisualSFM was primarily designed for visual images as input, a point cloud based on the thermal mouse data was successfully generated through camera calibration and image processing. In addition, the methods of (1) intensity transformation, (2) homogenization of the extrinsic landmarks by user input, and (3) a region-growing algorithm increased the number of feature points. As shown in Videos S1 and S2 the data of the different modalities can be displayed together and analyzed manually using the 3D THERMO-SCAN visualization tool. Here, a slice-by-slice visualization is possible that allows the examination of possible abnormalities in the CT slices with the external temperature distribution information (see Videos S1 and S2). For this analysis, an adaption of the color palettes was important to visually determine any anomalies in the temperature distribution of the anatomical thermal 3D model. As shown in Figure 11 (M3), different color palettes can easily be used and changed when creating the anatomical thermal 3D models.

Regarding the results of the SfM algorithm, not only could the number of camera positions needed be increased, but the numbers of points and projections could be significantly improved as well (see Table 1) using preprocessing. This can be explained by the fact that more features were found in the individual images due to the preprocessing, and it became more likely that these features would also be detected among the individual images. Adding the camera parameters obtained from camera calibration led to an increased number of points in the point cloud, and therefore an enhanced number of projections. In summary, the SfM algorithm could be improved appreciably by adding the camera information and preprocessing the thermal images.

A standard calibration grid (usually used for visual cameras) was applied for camera calibration. It was warmed by a heat lamp for recognition purposes. This is significantly simpler and more cost-effective than other methods—for example, the construction of a thermal calibration grid consisting of a heated metal and acrylic plate [24], using light bulbs as targets [25], or measuring a blackbody [26]. Even though the accuracy was lower in comparison, the approach used in this paper was demonstrated to be more than adequate for the current purpose. Regarding the extrinsic landmarks, preprocessing with computer vision algorithms helped to increase the number of features between images and thus improved the SfM results. Although this helped to define more features, especially in the low-contrast abdominal region of the mouse, one possibility for further research would be to place the extrinsic landmarks next to the mouse.

With reference to the limitations of the paper, it is necessary to consider several aspects. Even if the acquisition of the thermal data was sufficient for the process of the 3D model, the precision of the generated model should be increased—for example, by adding a hardware setup to ensure equidistant thermal images. Additionally, the resolution of the thermal camera limited the process, as the SfM algorithm gives better results with increasing resolution. The decision to use the FLIR E95 was a trade-off between image quality and ease of handling. This camera was valuable in the given setting within the animal experiment, for example, because it has an integrated battery and does not need to be connected to a computer during the acquisition. However, it will be important to consult other possible models with better resolution as alternatives in the future. Several working steps required manual execution to generate a combined anatomical and thermal model. With the help of the integration of camera calibration for the registration of the models, these steps might be automated in the future. Although the temperature distribution of the abdominal region within the anatomical thermal 3D models was satisfactory, the overlapping of the paws did not show perfect matching due to changes in position between the CT and thermal images’ acquisition. Even if the positioning of the animals (and therefore, the posture between acquisition of the CT and the thermal images) changed, the process could be successfully performed. However, this resulted in a less than ideal alignment of the paw region. This is negligible with regard to the overall goal of using a simulation to detect deep-seated inflammation and infections, which are more likely to be expected in the abdominal area.

Unlike similar approaches that have also generated thermal 3D models [5,7,16,18], it was possible to replace the third modality—a 3D scanner. Therefore, the anatomical thermal 3D models employed in this research were obtained from the complete set of CT image slices. During trials with 3D scanner apps, we discovered that the size of the test animals was too small to generate an accurate 3D model. Even if the use of a commercially available 3D scanner would solve this problem, the replacement of this third modality was cost-saving and more efficient. At the same time, the use of the CT data permitted us to improve the alignment of the outer shell and inner information. This resulted in the thermal images first being superposed with the 3D CT shell and then using a transformation of the reference system to register the thermal 3D shell with the anatomical data. With the additional information of internal conditions based on the CT data, this research goes one step further than the approaches of Chromy and Klima [5] and Grubišiü and Boš [7].

The thermal 3D shell presented in this paper is the starting point for a tool capable of simulating the inner temperature profile using skin temperature distribution as input. Based on the thermal properties of the respective tissues and anatomical information, a thermal model could be constructed, as done for human medicine in Mfolozi et al. [27]. For this purpose, the CT data would have to be classified according to the different tissues. Besides the segmentation of skin and bones, analysis of organ segmentation [28,29] as well as an extraction of possible tumors [30,31] have to be implemented. This thermal model could be based on the Pennes bioheat equation in addition to existing models, such as 2D thermal skin models [32,33].

Proceeding from this thermal model, a simulation of the inner temperature profile could, for example, be done using a Finite Element Method (FEM) approach, which was successfully used in Bayareh et al. [34] to simulate the temperature distribution of ulcers in the foot. Thus, based on the superficial heat distribution using thermal images, the internal temperature profile could be inferred with the help of the FEM simulation.

This is based on the fact that it has been shown that inflammation and infection can be detected using infrared thermography due to local changes of temperature based on variations in blood supply and metabolism, independent of fever [12]. This is supported by the correlation of the thermal temperature measurement with a established scoring method, as in the case of arthritis in a mouse model [14].

One application here could be the analysis of thermal images in 3D space, for which the anatomical thermal 3D models could provide a basis. In this way, to classify the thermal information, classical approaches or artificial intelligence methods, such as neural networks based on a feature extraction algorithm [35], could be used to detect pathological temperature distributions. These approaches could be applied to the planar thermal images generated by the data acquisition first and then be transferred to the 3D model. The advantage here would be that the thermal analysis would not be limited to an angle, a distance, and a certain area, as it is when using planar thermal images. Thus, a thermal analysis of the whole animal would be possible.

In addition, the superficial temperature distribution may reveal deep-seated infections. For example, in human medicine the superficial temperature distribution of the breast is directly related to the size of a tumor [36]. Additionally, the monitoring of therapy’s success regarding breast cancer has also been investigated using thermal images and FEM simulation [37]. Using the anatomical thermal 3D models presented in this research and the previously described FEM simulation, the otherwise superficial thermal analysis could be extended to the interior. With the help of this simulation, which tissue is affected by an infection could be deduced. At the same time, the spread of an infection induced within an animal experiment and the damage to surrounding tissue could be analyzed. It must be noted that heat conduction and heat convection (e.g., in the abdominal area) vary according to the organs underlying the regions of skin. Different organs and tissues have different thermal properties, such as heat conductivity and capacity, and density. Therefore, heat conduction and convection differ in different regions of the body.

Overall, both superficial and internal analyses of inflammation and infections could occur and be related. Simultaneously, longitudinal data could be analyzed to monitor the internal and external courses of the infection over time. The advantage here would be that the model represents the whole animal and not just a specific region. This would avoid having to vary the acquisition angle and distance between acquisition time points during longitudinal data collection, which would distort the analysis.

Thus, according to the 3R principle, the pain and associated stress of the animals could be reduced by the implementation of the previously described FEM simulation as a noninvasive complementary tool to detect deep-seated infections and inflammation. To achieve this goal, the drawbacks of the current study must be considered and potentially overcome in a future paper. That will include, for example, developing a hardware setup that allows the camera to be guided equidistantly around the animal. This could improve the quality of the data acquisition and the results of the SfM algorithm. To extend the applications based on this proof of concept, the next step would be to acquire data from animals with abdominal diseases, such as colitis. Based on the method presented in this research, anatomical thermal 3D models could also be generated with the newly acquired data from animals with diseases. The aims here would be to constantly improve the process algorithmically and to make it more relevant to practical applications with the help of automation. Transfer to human medicine applications would also be conceivable at this point, as the process presented here is also applicable to human data. The next milestone for the simulation would be to classify internal tissue based on anatomical information.

## 6. Conclusions

This paper demonstrated the process to generate anatomical thermal 3D models as a proof of concept. In our opinion, this is a necessary milestone for a later simulation of the internal temperature distribution and the detection of deep-seated infection and inflammation.

The current paper focused on the multimodal creation of a 3D model containing both the superficial temperature distribution and the internal anatomical information. The integrated thermal camera calibration and advanced image processing algorithms within an animal-based research application form the novelty of this paper.

Future work, such as a calculation of an inner thermal model and a simulation to predict the inner temperature profile could be integrated. At the same time, the process used to generate the anatomical thermal 3D models could be further automated to make the application more relevant for practical and clinical use. Additionally, these methodological approaches are also being applied to human studies regarding inflammation and infections as well. With the help of such a simulation and further analysis, deep-seated infections could be detected and analyzed more easily. This could allow earlier detection of infections and deterioration of condition in order to reduce animal suffering.

## Figures and Tables

**Table 1:** Comparison of VisualSFM parameters between original and preprocessed images with a camera.

	Number of Camera Positions	Points	Projections
Preprocessed Images (with camera information)	124 (100%)	1701	13,047
Preprocessed Images (without camera information)	124 (100%)	918	8078
Original Images	52 (42%)	137	1851

## Data Availability

Data sharing is not applicable to this article.

## Supplementary Materials

The following are available online at https://www.mdpi.com/1424-8220/21/4/1200/s1. Video S1: Final anatomical thermal 3D model of mouse M2 in the 3D-THERMO-SCAN visualization software (showing axial, sagittal, and coronal views). Video S2: Final anatomical thermal 3D model of mouse M3 in the 3D-THERMO-SCAN visualization software (showing axial, sagittal, and coronal views)

## Abbreviations

The following abbreviations are used in this manuscript:

CT	Computed tomography
GUI	Graphical user interface
SfM	Structure from motion
ROI	Region of interest
